# Unexpected massive bleeding during the modified LeFort III advancement surgery for Crouzon syndrome: A case report

**DOI:** 10.1002/ccr3.9001

**Published:** 2024-06-26

**Authors:** Zahra Sadat Modarresi, Narges Hajiani, Zeinab Bakhtiari, Farnoush Mohammadi

**Affiliations:** ^1^ Department of Oral and Maxillofacial Surgery, Dental School Tehran University of Medical Sciences Tehran Iran

**Keywords:** bleeding, case report, cranial sutures, craniofacial, Crouzon syndrome, von Willebrand disease

## Abstract

**Key Clinical Message:**

Vigilant monitoring for postoperative complications, including bleeding and dysrhythmia, is crucial in patients with craniosynostosis syndromes like Crouzon syndrome undergoing craniofacial surgery, with a thorough evaluation, including coagulation tests, assisting in diagnosing underlying conditions such as von Willebrand disease subtype 1 to inform appropriate management strategies.

**Abstract:**

Crouzon syndrome is a rare genetic disorder affecting craniofacial structures. Its etiology is the premature fusion of cranial sutures. The LeFort III advancement surgery is a commonly used approach to correct malformations related to midface hypoplasia. Complications following surgical treatment of craniosynostosis and craniofacial syndromes can include both intracranial and extracranial problems. Reporting of this syndrome and the surgery complications, in addition to consideration of other differential diagnoses, can help improve the treatment plan and surgery outcomes. The aim of the article is to report a 14‐year‐old female with Crouzon syndrome who underwent the modified LeFort III osteotomy and developed unexpected massive bleeding during the surgery. Post‐surgery, she experienced complications including dysrhythmia, hypothermia, and cyanosis. Treatment included fluid therapy, blood transfusions, and antibiotic therapy for suspected septic shock. Differential diagnosis was disseminated intravascular coagulation but was ruled out. Post‐discharge, coagulation tests suggested von Willebrand disease subtype 1 as the diagnosis. Excessive bleeding during surgery for craniosynostosis syndromes is a significant and concerning issue in the surgical management of Crouzon syndrome. For patients with von Willebrand disease who are candidates for elective surgeries, von Willebrand factor concentrates or recombinant von Willebrand factor can be used.

## 1 INTRODUCTION

Crouzon syndrome is a rare genetic disorder affecting craniofacial structures due to a mutation in one of the FGFR genes (*FGFR2* and *FGFR3*).^1^ The premature fusion of cranial sutures, including coronal and frontosphenoidal sutures and sphenoethmoidal synchondrosis in infants, causes the syndrome.^2^, ^3^ It affects about 1.6 per 100,000 people in the general population.^1^ This disorder can cause several craniofacial malformations such as proptosis, hypertelorism, and midface hypoplasia, as well as the relative prognathism of the mandible, orbital hypoplasia, strabismus, crossbite, and open bite.^1^, ^3^ The clinical findings of this syndrome can be categorized into extraoral signs (e.g., high and large forehead, flattening of the occipital region, maxillary hypoplasia, exorbitism with hypertelorism, and maxillary hypoplasia with mandibular prognathism) and intraoral signs (e.g., Class III occlusion, the maxillary dental arch in V shape, spaced teeth, and congenital cleft in the palate).^4^ Diagnosing Crouzon syndrome is through medical history and typical physical examination findings. Yet, in cases of spontaneous mutation with ambiguous clinical signs, genetic testing may be necessary. Further assessments, including brain imaging, may be needed to identify craniosynostosis or skeletal irregularities in specific conditions.^5^

The LeFort III advancement surgery was introduced in 1949 by Sir Harold Gillies to correct midface hypoplasia.^6^ The LeFort III advancement surgery is a commonly used approach to correct malformations related to the midface hypoplasia following 8 years of age.^7^ Complications following the surgical treatment of craniosynostosis and craniofacial syndromes can include both intracranial and extracranial consequences like infected and noninfected subgaleal hematoma, intracranial empyema, dural tear, cerebrospinal fluid leak with subgaleal collection, cerebral contusion, basal encephalocele, and granulomatous reaction to the absorbable material.^8^ Other possible complications include plate scarring through the skin, persistent craniolacuniae, non‐filiated hyperthermia, urinary infection, and several types of infectious (e.g., respiratory, ophthalmic, and central venous or mechanical intravenous line infections).^8^

As the prognosis of individuals with Crouzon syndrome relies on timely diagnosis and early treatment, reporting of the cases of this rare condition and its relevant treatment plans and outcomes are important. Moreover, reporting of this syndrome and the surgery complications with a focus on differential diagnoses can help improve the surgery outcomes. Therefore, the aim of the article is to report a 14‐year‐old girl with Crouzon syndrome who underwent the modified LeFort III osteotomy, developed unexpected massive bleeding during the surgery, and finally diagnosed with von Willebrand disease (VWD). The management, differential diagnoses, and postoperative and post‐discharge outcomes were also reported.

## 2 CASE HISTORY/EXAMINATION

**FIGURE 1:**
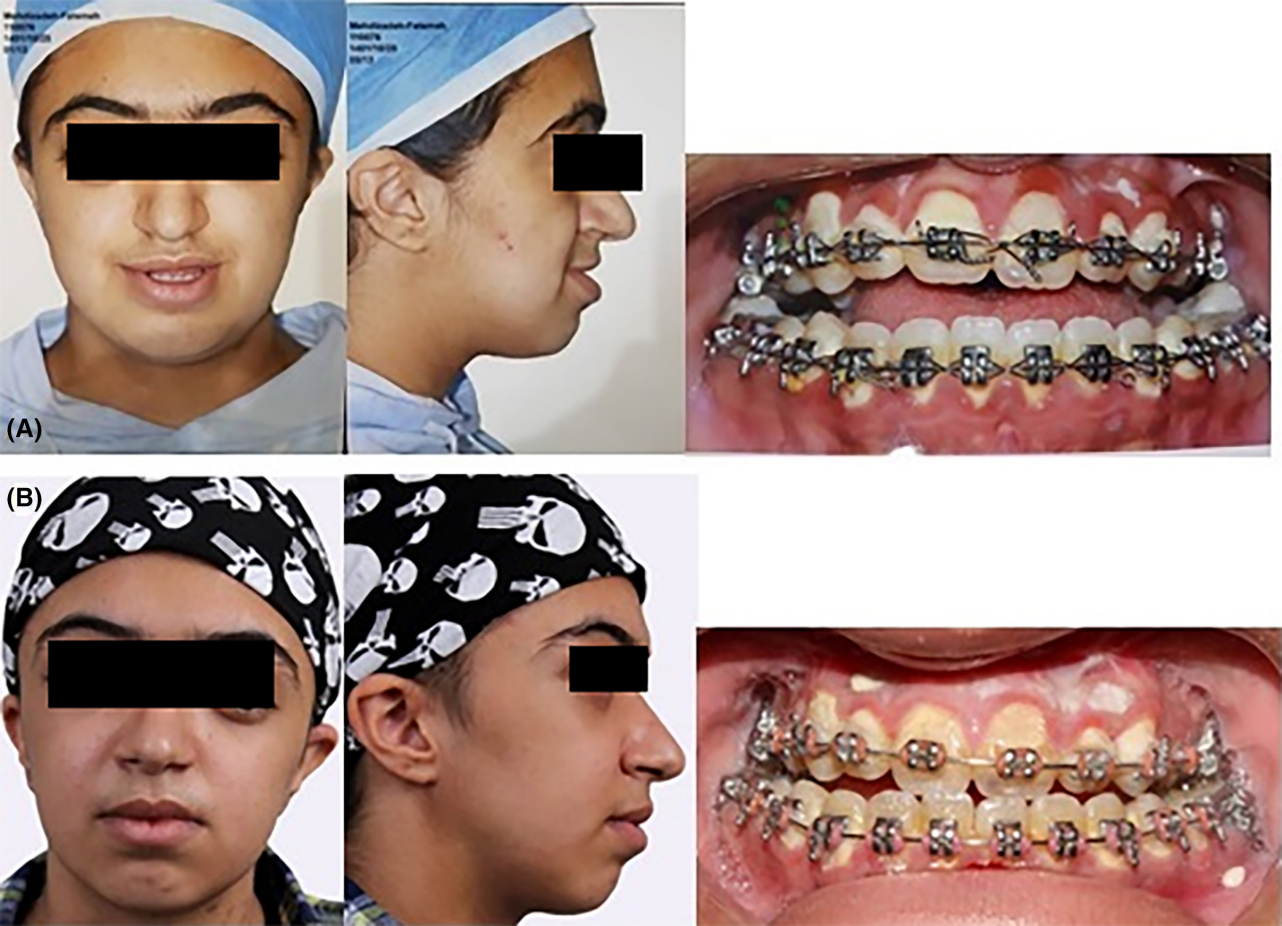
(A) Preoperative and (B) postoperative clinical photographs of the patient.

A 14‐year‐old female who had a known case of Crouzon syndrome was referred for treatment (Figure 1). She did not have any other known comorbidities like cardiovascular, gastrointestinal, neurological, or endocrinological diseases. The patient had not undergone any corrective surgeries for the craniofacial malformations caused by the syndrome. She did not have a family history of hematological disease. The patient did not use alcohol or cigarette smoking. A general physical examination showed no abnormal findings. She reported massive menstrual bleeding but did not experience abnormal bleeding after tonsillectomy and tooth extraction surgeries. To address the clinical symptoms, which included midface and orbit hypoplasia, the patient underwent a modified LeFort III osteotomy in our department.

## 3 METHODS

**FIGURE 2:**
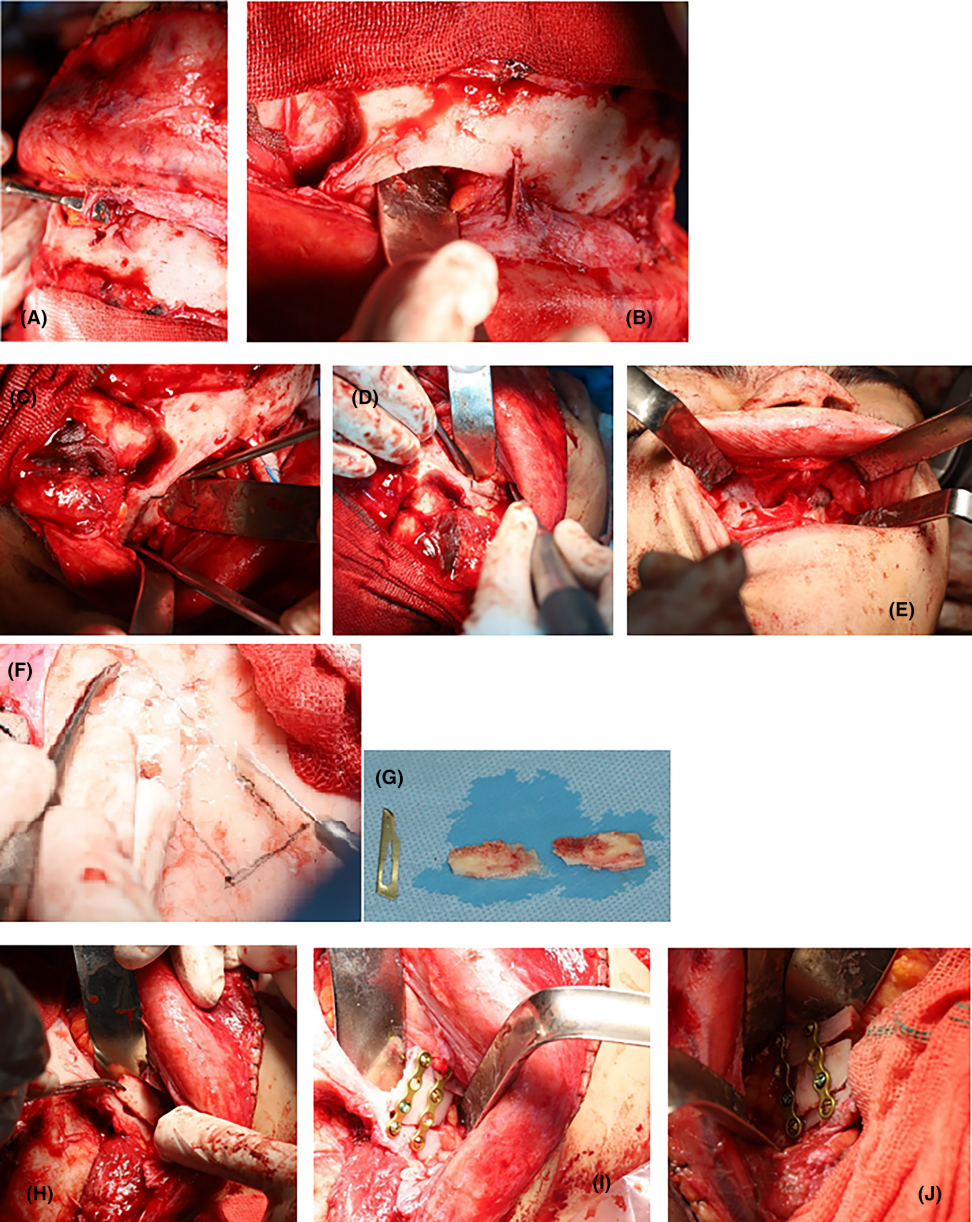
Intraoperative photographs of (A–E) flaps and osteotomies, (F–G) calvaria harvesting, and (H–J) lateral orbital rim graft.

The midface and orbit were advanced during the surgery to correct the malformations. To address the gap created in the lateral rim of the orbit after the advancing of the midface and orbit, a monocortical bone was taken from the calvaria and secured on both sides (Figure 2). However, during the surgery, an unexpected massive bleeding occurred with an estimated 1 L of blood loss. The bleeding was managed with local hemostatic agents, including oxidized regenerated cellulose, Gelfoam, and bone wax, in the operating room, and the patient was given two units of packed red blood cells to replace the blood loss.

## 4 CONCLUSION AND RESULTS

**FIGURE 3:**
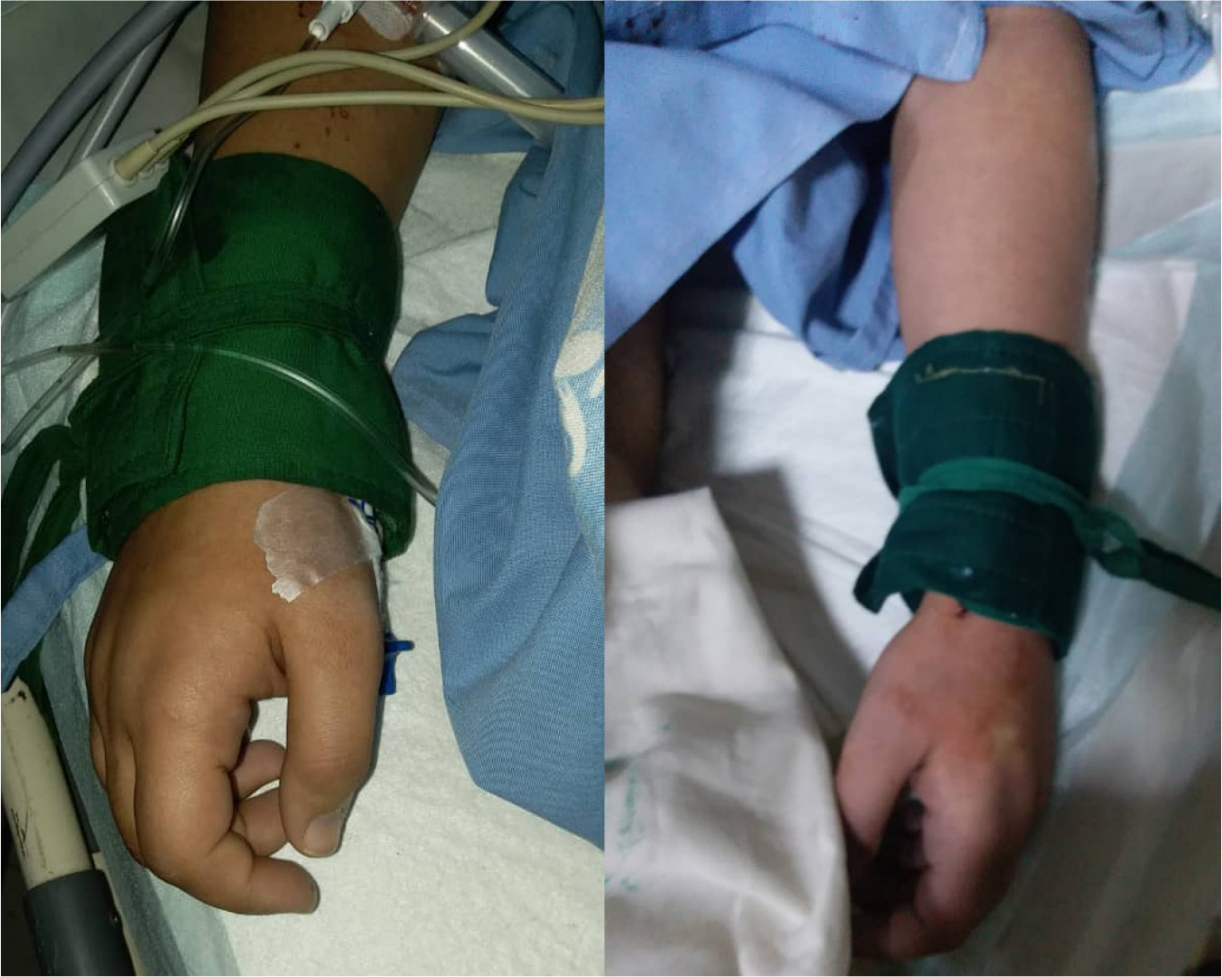
Upper limbs cyanosis in the case.

The patient was transferred to the intensive care unit (ICU) after the surgery. In the first hours after transferring the patient to the ICU, the patient developed dysrhythmia and tachycardia (heart rate: 120–170 beats per minute (bpm)). Her blood pressure dropped to 50/30 mmHg and her body temperature decreased to 33.7°C. Her left forearm and hand, as well as her right hand, got cyanotic (Figure 3). Following the hypotension and to prevent hemorrhagic shock, intravenous fluid therapy and three units of packed red blood cells were administered. During the blood transfusion, her heart rate decreased to 65 bpm and then rapidly increased to 160 bpm. After a few hours, the cyanosis in both hands resolved, and her blood pressure and heart rate got normal. The day after the surgery, her body temperature increased to 39.4°C.

Antibiotic therapy was initiated as the prophylaxis of septic shock. For this purpose, meropenem 2 g every 8 h and vancomycin 15 mg/kg every 12 h were administered. However, the results of the blood culture were negative. Due to the possibility of disseminated intravascular coagulation (DIC), the fresh frozen plasma (FFP) was administered, but due to the hypersensitivity reaction manifested with itching, urticaria, tachycardia, and hypotension, both antibiotics were discontinued 6 days post‐surgery.

The preoperative laboratory results revealed a white blood cell count of 6690 cells/mm^3^, platelet count of 415,000/mm^3^, hemoglobin level of 13.1 g/dL, prothrombin time of 14.7 s, partial thromboplastin time (PTT) of 38.1 s, international normalized ratio (INR) of 1.09, blood urea nitrogen of 10 mg/dL, creatinine level of 0.69 mg/dL, blood sugar of 99 mg/dL, erythrocyte sedimentation rate within normal limits, and C‐reactive protein level of 1 mg/dL. Two days after the surgery, her PTT increased to 99 s and the INR increased to 1.55. However, DIC was ruled out after checking the fibrinogen level, which was 239 mg/dL (normal range >150). On the third day after the surgery, the PTT decreased to 33 s and the INR to 1.24, and as they were in the normal range, the patient did not require FFP. The peripheral blood smear was evaluated for hemolysis and DIC, but there were no abnormal findings.

In postoperative evaluations, due to exposure of dura during calvaria harvesting, brain computed tomography (CT) was conducted, and no evidence of hydrocephalus was observed. Also, a color Doppler ultrasound of her upper limbs revealed normal flow in the arteries and right‐hand veins. However, the left cephalic and basilic veins showed no clear flow between the distal forearm and the middle of the arm. These results indicated the possibility of superficial venous thrombosis. Consultation with the pediatric cardiologist noted no abnormal findings in the cardiovascular system.

Following her discharge, coagulation tests were conducted at the National Blood Transfusion Organization as the referral center, and the results are presented in Table 1. Based on both the test outcomes and the history of bleeding issues, VWD subtype 1 was diagnosed. Follow‐up assessments included cephalometry post‐surgery to evaluate craniofacial changes and an ophthalmology examination to measure visual acuity and assess any changes in ocular parameters. In follow‐up, her diplopia improved. Imaging software was utilized to compare pre and postoperative photographs, enabling precise analysis of craniofacial alterations. Additionally, a series of three‐dimensional images were captured to provide a comprehensive view of the patient's facial structure before and after the surgical intervention. She was also referred to a hematologist/oncologist for further evaluation after discharge. Looking ahead, another orthognathic surgery, considered the second phase of her treatment, was planned to further enhance her facial relationships.

**TABLE 1:** Laboratory test results of the patient.

Test	Result	Unit	Method	Normal ranges
Factor VIII:C	71	%	One‐stage assay	43–155
Factor II	111	%	One‐stage assay	48–119
aPTT	34	Sec	Clotting time	24.6–38.4
Factor IX	109	%	One‐stage assay	60–138
aPTT control	30	Sec	Clotting time	—
Factor V	84	%	One‐stage assay	62–125
PT	11.5	Sec	Clotting time	10–14.1
Factor VII	102	%	One‐stage assay	55–133
PT control	11	Sec	Clotting time	—
Factor X	103	%	One‐stage assay	64–131
Factor XI	122	%	One‐stage assay	55–139
VWF activity (VWF:GPIbM)	37	%	Immunoassay	50–200
VWF antigen (VWF:Ag)	41	%	Immunoassay	46–153
VWF collagen binding (VWF:CB)	40	%	ELISA	50–160
VWF:RCo/VWF:Ag	0.9	Ratio	Ratio	>0.6: Positive type 1 VWD <0.6: Positive type 2 VWD
VWF:CB/VWF:Ag	1	Ratio	Ratio	>0.6: Normal

Abbreviations: GPIbM, glycoprotein Ib multimer; PT, prothrombin time; PTT, partial thromboplastin time; VWD, von Willebrand disease; VWF, von Willebrand factor.

## 5 DISCUSSION

In this case report, a girl with Crouzon syndrome underwent surgery and massive intraoperative bleeding occurred. Excessive bleeding during surgery for craniosynostosis syndromes is a significant and concerning issue in the surgical management of these patients. In our case, VWD was diagnosed through laboratory tests. It is essential to pay attention to blood loss amount and coagulation problems during surgery for craniosynostosis syndromes. For the patient, differential diagnoses of sepsis, DIC, hemolysis, and volume overload were considered.

Early diagnosis and treatment of craniosynostosis syndromes are crucial in preventing brain growth restriction caused by distortion and insufficient cranium volume.^9^ The treatment of Crouzon syndrome typically involves two stages: (1) during the first few years of life, surgical intervention is mainly employed to release the fused sutures and perform cranial decompression and reshaping and (2) after the age of 6 years, reconstruction surgery is recommended to correct craniofacial deformities, including midface deficiency, Class III malocclusion, and other anomalies that require surgery.^9^, ^10^, ^11^

The modified LeFort III osteotomy can be used as a treatment option for midface deficiencies when the nasomaxillary complex is not involved. The study by Tessier achieved positive results using this technique, which remains a viable treatment choice.^12^ In our case who was a 14‐year‐old girl who underwent the modified LeFort III advancement surgery, an unexpected intraoperative massive bleeding occurred. Craniosynostosis surgery for children often results in significant blood loss and that might require blood transfusions. However, with the advancement of surgical techniques and improved care, the morbidity and mortality rates associated with craniofacial surgeries have decreased.^13^ Nevertheless, blood loss during this procedure can vary significantly, ranging from 20% to 500% of blood volume. This wide range has a substantial risk for complications associated with blood transfusion, including infection, allergic reactions, and the potential for inappropriate blood transfusion.^14^

There are several differential diagnoses for Crouzon syndrome, including Apert syndrome, Muenke syndrome, Pfeiffer syndrome, and nonsyndromic craniosynostosis which refer to distinct conditions involving cranial abnormalities, whereas differ in their specific characteristics and genetic origins.^5^ The hallmark of Apert syndrome is craniosynostosis and symmetric syndactyly of the hands and feet,^4^ while our case did not present symmetric four‐limb syndactyly. In Muenke syndrome, there are temporal bossing, craniosynostosis, macrocephaly, and facial asymmetry. In particular, patients with Muenke syndrome have sensorineural hearing loss.^15^ This is in contrast with the presentations of our reported case. In our case, the intraoperative bleeding and coagulation tests which were performed due to the massive bleeding, led to a high probability diagnosis of VWD. VWD has a prevalence between 108.9 and 2200 per 100,000 in the general population and is presented with bleeding in more than 70% of cases.^16^ The most common symptoms in patients with VWD include mucocutaneous bleeding, massive menstrual bleeding, easy bruising, epistaxis, bleeding after dental or surgical procedures, and massive bleeding from wounds.^17^ Its diagnosis is based on both abnormal laboratory tests and a hemorrhagic phenotype.^18^ von Willebrand factor (VWF) is classified into three subtypes depending on the cause of its dysfunction, which can be due to a deficiency or a qualitative impairment in its function. In subtype 1, there is a partial factor deficiency, whereas subtype 3 involves a complete factor deficiency. A decrease in factor quality characterizes subtype 2. The latest guidelines suggest that patients with a VWF level below 50 international unit (IU)/dL and a history of bleeding should be diagnosed with VWD. Subtype 1 is the most prevalent subtype, accounting for 70%–80% of cases.^17^ In patients with VWD and massive bleeding or those who are undergoing elective surgeries, plasma‐derived VWF concentrates with or without Factor VIII or recombinant VWF can be used.^17^, ^18^

In conclusion, we have reported a patient who experienced massive bleeding during the modified LeFort III advancement surgery for Crouzon syndrome. Blood loss during surgeries for craniosynostosis syndromes can be significant and should be considered by surgeons and anesthesiologists. Coagulation disorders can exacerbate bleeding complications during surgical procedures. To prevent the recurrence of bleeding in these patients, various prophylactic methods should be used before elective surgeries. In our case, after the surgery, the patient was diagnosed with a VWD through laboratory tests. For patients with VWD who are candidates for elective surgeries, VWF concentrates or recombinant VWF can be used. This study highlights the fact that several types of differential diagnoses in patients with Crouzon syndrome who presented with massive bleeding should be considered. In summary, thorough preoperative assessment and intraoperative management in patients with syndromic craniofacial anomalies, close postoperative monitoring for complications, comprehensive coagulation testing before major surgeries, and a multidisciplinary approach involving surgical, hematologic, and critical care specialists to ensure optimal patient care and outcomes are suggested.

## AUTHOR CONTRIBUTIONS

**Zahra Sadat Modarresi:** Conceptualization; data curation; investigation; methodology; resources; validation; visualization; writing – original draft; writing – review and editing. **Narges Hajiani:** Data curation; investigation; resources; writing – original draft; writing – review and editing. **Zeinab Bakhtiari:** Writing – original draft; writing – review and editing. **Farnoush Mohammadi:** Conceptualization; resources; supervision; writing – original draft; writing – review and editing.

## FUNDING INFORMATION

The authors received no financial support for this research.

## CONFLICT OF INTEREST STATEMENT

The authors declare that they have no conflict of interests.

## ETHICS STATEMENT

Written informed consent was obtained from the patient to publish this report in accordance with the journal's patient consent policy.

## CONSENT

Written informed consent was obtained from the patient to publish this report in accordance with the journal's patient consent policy.

## Data Availability

The data that support the findings of this study are available on request from the corresponding author. The data are not publicly available due to privacy or ethical restrictions.
